# Higher proliferation of peritumoral endothelial cells to IL-6/sIL-6R than tumoral endothelial cells in hepatocellular carcinoma

**DOI:** 10.1186/s12885-015-1763-2

**Published:** 2015-11-02

**Authors:** Peng-Yuan Zhuang, Jian-Dong Wang, Zhao-Hui Tang, Xue-Ping Zhou, Zhi-Wei Quan, Ying-Bin Liu, Jun Shen

**Affiliations:** Department of General Surgery, Xinhua Hospital, School of Medicine, Shanghai Jiao Tong University, 1665 Kong jiang Street, Shanghai, 200082 China

**Keywords:** Peritumoral endothelial cell, Tumor endothelial cell, IL-6, sIL-6R, gp130

## Abstract

**Background:**

This study aimed to explore the responses to the interleukin-6 (IL-6)/soluble interleukin-6 receptor (sIL-6R) complex in peritumoral endothelial cells (PECs) and tumor endothelial cells (TECs), as well as determine the signaling pathways in the angiogenesis of hepatocellular carcinoma (HCC).

**Methods:**

The expression of IL-6, IL-6R, gp130, CD68, HIF-1α, and microvessel density (MVD) were assessed with an orthotopic xenograft model in nude mice. ECs were incubated under hypoxic conditions to detect IL-6 and gp130. The proliferation of PECs and TECs in the presence of IL-6 and sIL-6R, as well as the expression of gp130, JAK2/STAT3, PI3K/AKT in endothelial cells were measured.

**Results:**

Peritumoral IL-6, IL-6R, gp130, CD68, and HIF-1α expression, as well as MVD, gradually increased during tumor growth. Hypoxia could directly induce IL-6 expression, but not gp130 in PECs. The co-culture of IL-6/sIL-6R induced much higher PEC proliferation and gp130 expression, as well as the elevated phosphorylation of JAK2 and STAT3, however not the phosphorylation of PI3K and AKT.

**Conclusions:**

PECs exhibited higher proliferation in response to IL-6/sIL-6R co-treatment compared with TECs in HCC via the up-regulation of gp130 /JAK2/STAT3. PEC and its associated peritumoral angiogenesis microenvironment may be a potential novel target for anti-angiogenic treatment.

**Electronic supplementary material:**

The online version of this article (doi:10.1186/s12885-015-1763-2) contains supplementary material, which is available to authorized users.

## Background

Angiogenesis is a fundamental event in the process of hepatocellular carcinoma (HCC) growth and metastatic dissemination [1, 2]. The current understanding of angiogenesis is mostly focused on tumor angiogenesis. However, reports from our group and others have implied that more pro-angiogenic factors are present in pertitumoral liver tissue compared with those in tumor tissue [3–7]. Furthermore, peritumoral angiogenesis can predict tumor metastasis and patient prognosis [6, 8, 9]. Therefore, the activation and proliferation of endothelial cells (ECs) to develop new vessels from pre-existing ones are vital for the process of angiogenesis. ECs may be strictly classified into tumor ECs (TECs) and peritumoral ECs (PECs). TECs and PECs should be specifically studied in terms of their different biological functions during angiogenesis and response to pro-angiogenic factors.

TECs and PECs are known to possess different characteristics in terms of their morphology [10], pathophysiology [11], cytogenetics [12], epigenetics [13], and gene expression [14]. Our previous study showed that TECs demonstrate increased apoptotic resistance, motility, and other pro-angiogenic properties, with the increased ability to adhere to tumor cells and survive in the tumor environment, compared with PECs [15]. These results suggested that the differences between PECs and TECs may account for their different levels of efficiency in therapy, such as increased resistance to adriamycin, 5-fluorouracil, and sorafenib in TECs [15]. Therefore, an improved understanding of the different signaling events that mediate the response to pro-angiogenic factors between TECs and PECs will lead to the development of improved anti-angiogenesis therapies for HCC.

Interleukin-6 (IL-6) plays a role in primary tumor progression [16]. The genetic signature derived from non-tumor liver tissue may reflect the promoting effects of IL-6 on the development of metachronous tumors that are independent from the primary resected HCC [17, 18]. Tumor-derived IL-6 is known to promote EC proliferation and new blood vessel generation that support the high metabolic demands of tumor cells. The cell signaling pathway of IL-6 for EC proliferation has been previously studied, and the formation of the IL-6/IL-6R/gp130 complex on the EC surface can induce PI3K/AKT or JAK2/STAT3 activation and cell proliferation [19, 20]. ECs lack IL-6R, and soluble IL-6R eventually plays an important role in EC proliferation via the trans-signaling pathway; consequently, signaling occurs after the formation of the IL-6/sIL-6R/gp130 complex on the EC surface [21, 22]. However, the aforementioned functional studies were mostly conducted with established cell lines, such as the human umbilical vein ECs (HUVECs). The use of HUVECs causes an obvious disadvantage because these cells are derived from large vessels that lack the typical characteristics of TECs and PECs. Therefore, the characterization of different responses to IL-6/sIL-6R treatment in TECs and PECs, as well as their respective signaling pathways, may reveal EC characterization.

A prevalent paradigm in tumor biology is that TECs may have a much higher proliferation rate when responding to pro-angiogenic factors, such as IL-6 or sIL-6R. This paradigm was challenged by the present study. Our results showed that PECs had much higher levels of cell proliferation when co-cultured with IL-6/sIL-6R by automatically up-regulating their cell surface gp130 levels *in vitro* and activating JAK2/STAT3 signaling pathway, which had no effect on the PI3K/AKT signaling pathways. Our results also demonstrated that IL-6 secretion by PECs was induced by peritumoral hypoxia, and the increased IL-6 levels contributed to the much higher proliferation of the cells.

## Methods

### Cell lines and culture conditions

PECs and TECs were obtained from surgical HCC specimens and their surrounding normal liver tissue immediately after removal from patients, as previously described in our laboratory [15]. ECs were isolated from the cell suspension by anti-CD105 antibodies coupled to magnetic beads (Miltenyi Biotech, Germany) and magnetic cell sorting with the MACS system (Miltenyi Biotech,, Germany) [15]. Cells were grown in complete EBM-2 medium supplemented with 10 % fetal bovine serum, 100 U/mL penicillin, and 100 μg/mL streptomycin. ECs between passages 1 and 6 were used in the subsequent experiments within six months after resuscitation. The study was approved by the Xinhua Hospital Research Ethics Committee. Informed consent was obtained according to the committee's regulations.

### Tumor xenograft

Six-week-old male BALB/c nu/nu nude mice, weighing approximately 20 g each (Shanghai Institute of Materia Medica, Chinese Academy of Sciences, Shanghai, China), were housed in laminar flow cabinets under specific pathogen-free conditions. Orthotopic implantation of human HCC cell line HCCLM3 in BALB/c mice was performed as previously described [23–25]. The mice were cared and handled in accordance with the National Institutes of Health Guidelines for the Care and Use of Laboratory Animals. The experimental protocol was approved by the Shanghai Medical Experimental Animal Care Committee. To study EC proliferation and angiogenesis-related genes in peritumoral tissue during tumor growth, 18 mice of the HCC tumor model was established by orthotopic implantation of a histologically intact tumor tissue derived from HCCLM3 cell line, and 6 mice were killed at the end of the 2, 5 and 7wk after tumor injection respectively.

### Tissue microarray and immunohistochemistry (IHC)

We constructed a tumor microarray (Shanghai Biochip Co., Ltd, Shanghai, China) as described in our previous study [9]. After reviewing HE-stained slides for the location of tumor tissue and tissue adjacent to tumor (TAT) with the distance of 2 cm away from tumor, we constructed TMA slides (collaborated with Shanghai Biochip Company, Ltd, Shanghai, China). Two cores were taken from each formalin-fixed, paraffin-embedded HCC and TAT sample, respectively, by using punch cores that measured 1.0 mm in greatest dimension from the nonnecrotic area of tumor foci and TAT. The following primary antibodies were used: mouse monoclonal anti-IL-6, anti-IL-6R, and anti-gp130 antibodies (Abcam, Cambridge, MA, USA), rabbit polyclonal anti-CD31 and anti-HIF-1α antibodies (Abcam, Cambridge, MA, USA), and mouse monoclonal anti-CD68 antibody (1:100; Zymed Laboratories, San Francisco, CA, USA). The density of positively stained cells was measured as specified in our previous study [9]. The integrated optical density was determined using Image-Pro Plus v6.2 software (Media Cybernetics, Inc., Bethesda, MD, USA) with the same setting for all the slides. To quantify the mean microvessel density (MVD), five fields at 100× magnification in the CD31-stained “hotspot” were photographed. The MVD was quantified as the ratio of the CD31-positive area to the total area.

### Reverse transcription PCR (RT-PCR) analysis

The mRNA levels of IL-6, IL-6R, and gp130 in the peritumoral liver tissue, as well as those of IL-6 and gp130 in the PECs and TECs, were detected as follows. Total RNA was extracted following the manufacturer’s protocol (Invitrogen). Real-time RT-PCR analysis for quantification was performed using an SYBR Premix Ex Taq™ (perfect real time; TaKaRa). The relative mRNA expression was normalized to that of β-actin. The relative amount of tissue mRNA was standardized by the amount of β-actin mRNA, and expressed as − △CT = CT(factor) − CT(β-actin). The ratio of the number of mRNA copies to the number of β-actin mRNA copies was then calculated as 2^–△CT^ × K, where K is a constant. The primers for RT-PCR are listed in Additional file 1: Table S1.

### Western blot analysis

After 24 and 48 h of incubation with different stimulatory agents (IL-6, sIL-6R, IL-6 and sIL-6R), the PECs and TECs were harvested and lysed or homogenized for Western blot analysis. The primary antibodies for the analysis included IL-6, IL-6R, gp130, p-JAK2 (Y1007/1008), JAK2, p-STAT3(Y705), STAT3, p-PI3K(Y607), PI3K, p-Akt(S473), Akt and GAPDH (Cell Signaling Technology, Beverly, MA). Specific protein expression levels were normalized to the GAPDH protein. All experiments were performed in triplicate and densitometric analysis was performed for the statistical significance.

### Immunocytochemistry

The primary antibodies used were rabbit polyclonal gp130 (1:100; Santa Cruz, CA, USA) and rabbit monoclonal IL-6 (1:200; Abcam, Cambridge, MA, USA) antibodies. The primary antibody was detected by the secondary antibodies of anti-rabbit IgG-TR (Texas Red; Abcam) and anti-mouse IgG-FITC (Abcam). Cells were incubated with the respective primary antibody overnight at 4 °C. Sections were washed three times in PBS, followed by incubation with the secondary antibody for 1 h at room temperature. Samples were analyzed with an inverted fluorescence microscope (Olympus IX51) equipped with an Olympus Qcolor 3 digital camera (Olympus).

### Cell proliferation assay

The cultured PECs or TECs were plated in triplicate into 96-well plates containing EBM-2 medium for 10 h. To detect the cellular response to IL-6 (20 ng/ml) and sIL-6R (100 ng/ml) treatment (R&D Systems, Minneapolis, MN, USA), cells were cultured for 24, 48, and 72 h in medium containing different drugs at the indicated concentrations. The Cell Counting Kit-8 (CCK-8) (Dojindo Laboratories, Kumamoto, Japan) was used to determine cell viability. All experiments were performed in triplicate.

### Cell culture and hypoxia

PECs and TECs were incubated in 37 °C under a humidified atmosphere (5 % CO_2_) for 24 and 48 h in the presence or absence of cobalt chloride (CoCl_2_, 100 μmol/L), which was used to mimic the effects of hypoxia by its minimal effect on cell viability Both cell types were then harvested for detection of IL-6 and gp130 expression by immunocytochemistry and RT-PCR.

### Statistical analysis

All statistical analyses were performed with SPSS version 16.0 software. The Spearman rank correlation coefficient was used to analyze the correlations among parameters. Quantitative variables were expressed as the mean ± standard deviation, and analyzed with the *t*-test. *P* < 0.05 was considered statistically significant.

## Results

### Gradually increased levels of IL-6 IL-6R, gp130, CD68, and MVD in peritumoral liver tissue

**Fig. 1:**
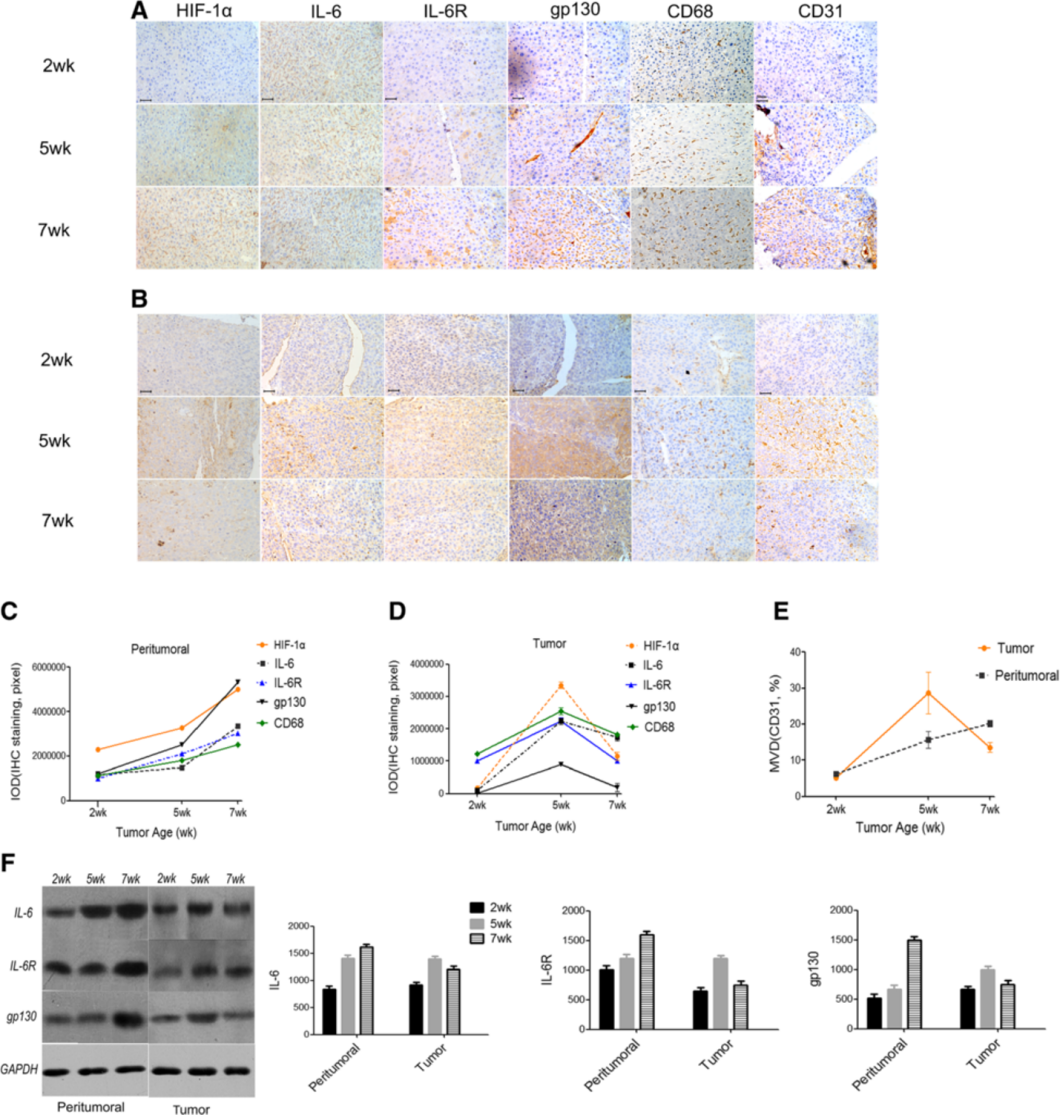
**a**–**f** levels of IL-6, IL-6R, gp130, CD68(+) macrophages and MVD in peritumoral liver tissue and tumor tissue. Peritumoral HIF-1α, IL-6, IL-6R, CD68, gp130, and CD31 gradually increased after seven weeks of tumor growth. The samples were then examined by tissue microarray and IHC (expression levels from representative mice in the seven weeks of tumor growth, as indicated by IHC, are shown in **a**; the expression curve during the seven weeks is shown in **c**). However, the expression patterns of all the six factors were not similar in the tumor tissue because they were gradually up-regulated until five weeks and then became down-regulated, the expression curves after seven weeks of tumor growth are shown in **b** and **d**. The expression of IL-6, IL-6R, and gp130 in both peritumoral and tumor tissue were also confirmed by Western blot analysis (**f**). Bar, 50 μm. * P < 0.05; **P < 0.01

Compared with HCC tumor tissue, several EC proliferation and angiogenesis-related genes in peritumoral tissue were gradually up-regulated during seven weeks of tumor growth, including IL-6, IL-6R, and gp130, which was confirmed by RT-PCR (using mouse-specific primers), IHC staining (Fig. 1a, c) and Western blot (Fig. 1f). The results showed more than a twofold change in the expression ratio of seven weeks/five weeks. Specifically, the expression of *IL-6* (3.45-fold), *IL-6R* (2.35-fold), and *gp130* (4.21-fold) changed. The expression levels of representative mice after seven weeks of tumor growth were visualized by IHC (Fig. 1a), and the corresponding expression curve after seven weeks was shown in Fig. 1c. The Western blot analysis also revealed the similar trend of IL-6, IL-6R and gp130 during tumor growth (Fig. 1f). Peritumoral MVD followed a similar trend after seven weeks of tumor growth, which was confirmed by IHC (20.2 % ± 0.9 % *vs*. 15.6 % ± 2.4 % for seven and five weeks, respectively; *P* = 0.021, Fig. 1e). Previous studies stated that macrophages contribute to sIL-6R secretion [26, 27]. We found that peritumoral CD68(+) macrophages were also up-regulated on the seventh week of tumor growth (Fig. 1a); this change was significantly associated with peritumoral IL-6R (rr = 0.556, *P* = 0.001).

The expression pattern of all the factors (IL-6, IL-6R, gp130, and CD68) was not similar among the tumor tissues, such that all the factors were gradually up-regulated until five weeks. Subsequently, all the factors were down-regulated, and RT-PCR indicated that the expression ratios of seven weeks/five weeks of *IL-6*, *IL-6R*, and *gp130* in tumor growth decreased by 0.43-fold, 0.47-fold, and 0.33-fold, respectively. These results were also confirmed by IHC (Fig. 1b, d) and Western blotting (Fig. 1f). Similarly, the MVD was down-regulated from that after five weeks of tumor growth (13.5 % ± 1.4 % *vs*. 28.6 % ± 5.8 % for seven and five weeks, respectively; *P* = 0.014; Fig. 1d). These results were much lower than that of peritumoral MVD (*P* = 0.004).

### Hypoxia induced IL-6 expression in PECs

**Fig. 2:**
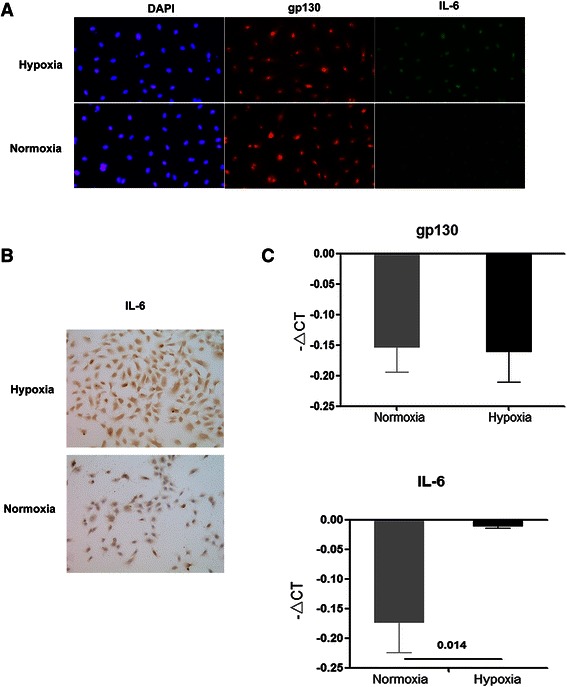
**a**–**d** Hypoxia induced IL-6 expression in PECs. Hypoxia could significantly and directly up-regulate the expression of IL-6 after 48 h in PECs, as indicated by immunocytochemistry (**a**), IHC (**b**), and RT-PCR (**c**). However, hypoxia had no direct effects on gp130 expression in PECs, as indicated by immunocytochemistry (**a**) and RT-PCR (**c**). Bar, 50 μm

To further elucidate the peritumoral microenvironment during the up-regulation of IL-6, we stained peritumoral tissues with the hypoxia indicator HIF-1α. The peritumoral expression of IL-6 gradually increased during tumor growth (Fig. 1a, c). Peritumoral HIF-1α were in accordance with the peritumoral IL-6 expression (rr = 0.721, *P* = 0.004). To determine whether hypoxia can directly up-regulate the expression of IL-6 in PECs, we treated PECs to hypoxic or normoxic conditions for 24 and 48 h. The results demonstrated that IL-6 was significantly up-regulated in PECs under hypoxic conditions for 48 h, which was confirmed by immunocytochemistry (Fig. 2a), IHC (Fig. 2b), and RT-PCR (mean − △CT, −0.01 ± 0.003 *vs*. –0.17 ± 0.052 for hypoxic and normoxic conditions, *P* = 0.014, Fig. 2c). However, we found that hypoxia had no direct effect on PEC proliferation *in vitro* for 24 and 48 h (1.17 ± 0.01 *vs*. 1.16 ± 0.01, *P* = 0.714 and 2.23 ± 0.01 *vs*. 2.25 ± 0.02, *P* = 0.628 under hypoxic and normoxic conditions for 24 and 48 h, respectively; cell proliferation was evaluated by the CCK-8 assay).

### Hypoxia did not up-regulate gp130 in PECs

PECs were further characterized for the distinct expression of endothelial surface markers: IL-6R(−) and gp130(+). The expression levels of endothelial markers were tested at the first, third, and fifth passages, and similar results were confirmed during cell culture. We investigated the expression levels of gp130 in PECs under hypoxic conditions. The levels of gp130 were not affected by hypoxia for 24 and 48 h based on immunocytochemistry (gp130 expression under hypoxic and normoxic conditions for 48 h was shown in Fig. 2a) and RT-PCR [mean − △CT: −12.5 ± 0.04 *vs*. −13.1 ± 0.05 (*P* = 0.825) and −10.7 ± 0.05 *vs*. −9.9 ± 0.07 (*P* = 0.545) under hypoxic and normoxic conditions for 24 and 48 h, respectively, Fig. 2c].

### Effect of IL-6 and sIL-6R on the proliferation of PECs and TECs

**Fig. 3:**
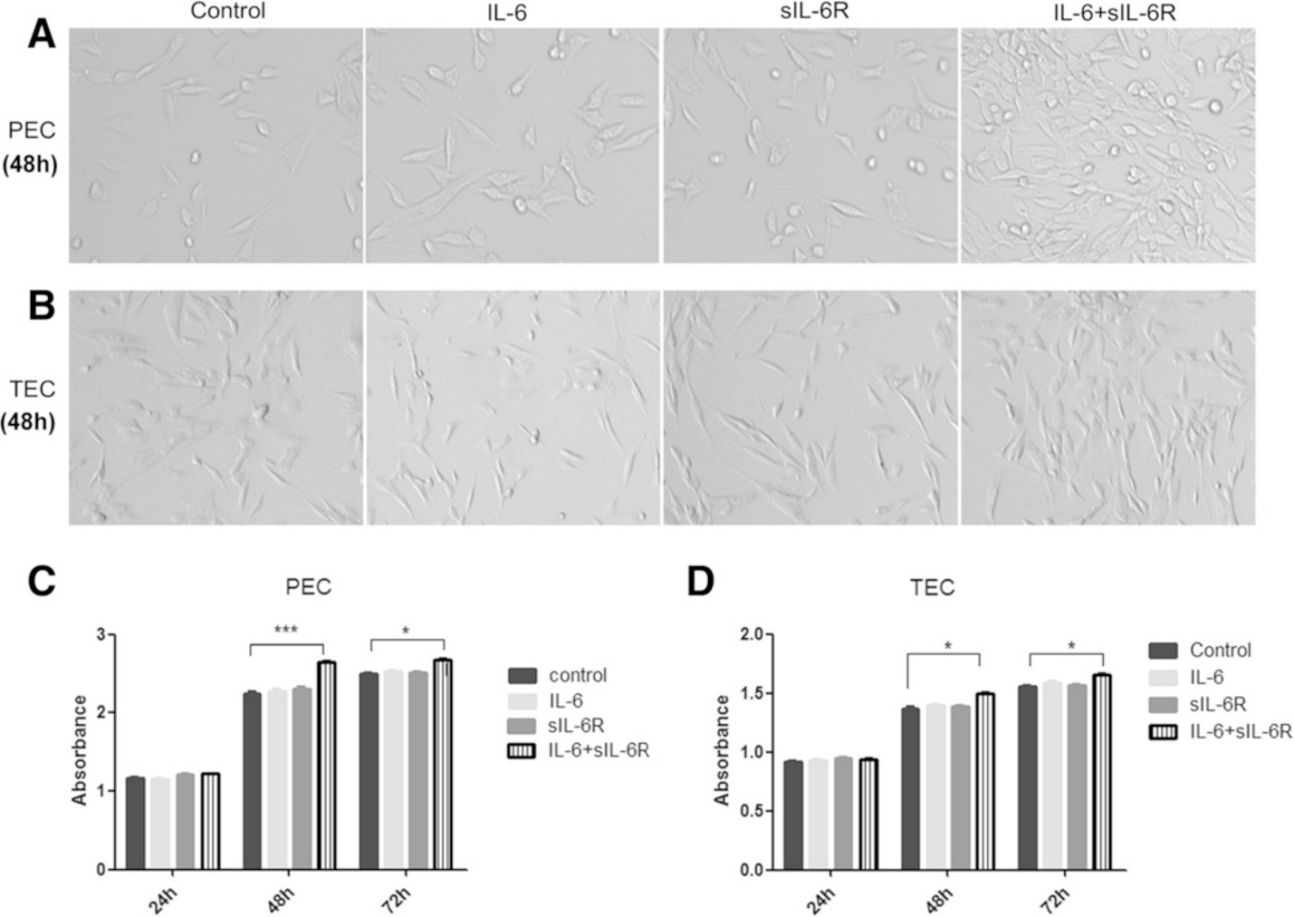
**a**–**d** Effect of IL-6 and sIL-6R on the proliferation of PECs and TECs. IL-6 or sIL-6R alone had no effect on the proliferation on PECs and TECs. The co-culture of both IL-6 and sIL-6R could induce PEC and TEC proliferation. PECs showed much higher proliferation in response to the co-cultured environment for 48 and 72 h as compared with TECs. Representative images of PECs (**a**) and TECs (**b**) in different culture conditions after 48 h. Absorbance of PECs (**c**) and TECs (**d**) at 24, 48, and 72 h in different culture conditionss

We subsequently determined the effects of IL-6 and sIL-6R on EC proliferation, and found that neither IL-6 nor sIL-6R alone had an effect on the proliferation of PECs and TECs (Fig. 3). To determine whether the presence of both IL-6 and sIL-6R can induce the proliferation of PECs and TECs, we co-cultured both cell types with IL-6 and sIL-6R. Interestingly, the proliferation of both PECs and TECs was much higher when the cells were grown together with IL-6 and sIL-6R compared with that of cells grown with either IL-6 or sIL-6R for 48 h and 72 h (especially predominant for 48 h), and no obvious different was observed in 24 h. Therefore, cell proliferation increased in response to the co-culture environment. Further, PECs showed a much higher rate of proliferation in response to the co-culture environment than TECs [2.65 ± 0.01 *vs.* 2.25 ± 0.02 for PECs under the co-cultured and vehicle conditions for 48 h, respectively (*P* = 0.0004); 1.50 ± 0.01 *vs.* 1.37 ± 0.01 for TECs under the co-cultured and vehicle conditions for 48 h, respectively (*P* = 0.029)]; cell proliferation was evaluated by the CCK-8 assay; Fig. 3, Table 1].

**Table 1:** The absorbance of ECs under different culture conditions of IL-6 and/or sIL-6R

		Control	IL-6	sIL-6R	IL-6 + sIL-6R
TECs	24 h	0.92 ± 0.01^a^	0.93 ± 0.01	0.95 ± 0.01	0.94 ± 0.01
	48 h	1.37 ± 0.01	1.40 ± 0.01	1.39 ± 0.01	1.50 ± 0.01*
	72 h	1.56 ± 0.01	1.59 ± 0.02	1.57 ± 0.01	1.66 ± 0.01*
PECs	24 h	1.16 ± 0.01	1.15 ± 0.01	1.21 ± 0.01	1.22 ± 0.01
	48 h	2.25 ± 0.02	2.28 ± 0.02	2.31 ± 0.01	2.65 ± 0.01***
	72 h	2.50 ± 0.01	2.53 ± 0.01	2.51 ± 0.02	2.71 ± 0.01*

^a^cell proliferation was evaluated by CCK-8 assay and results were expressed as the absorbance of each well which was read at 490 nm with a microplate reader (Nexcelom, Lawrence, MA). Culture medium (EBM-2 medium) without cells was used as blank control. All experiments were performed in triplicate

Student *t* test was conducted between different groups with stimulation of (*IL-6 and /or sIL-6R)* and control group

* P < 0.05; *** P < 0.001

### Effects of IL-6 or sIL-6R on the expression of gp130 and JAK2/STAT3, PI3K/AKT in PECs and TECs

**Fig. 4:**
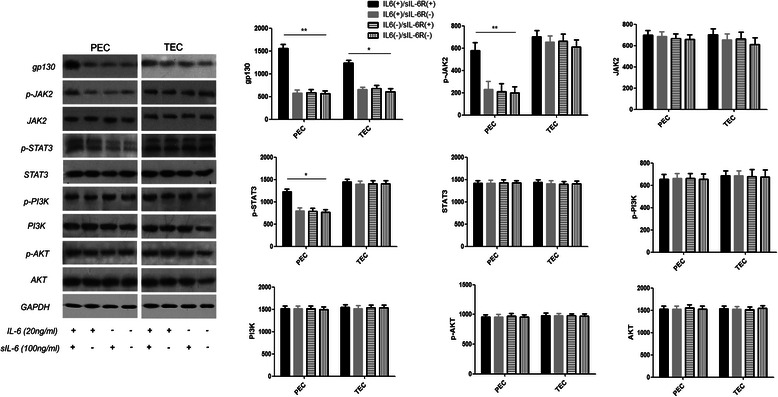
Effect of IL-6 or sIL-6R on the expression of gp130 and JAK2/STAT3, PI3K/AKT in PECs and TECs. Administration of IL-6 or sIL-6R alone could not promote the expression of gp130, PI3K, and AKT after 24 and 48 h. However, co-culture with both IL-6 and sIL-6R for 48 h could lead to a synergistic increase in gp130 levels, and the increased phosphorylation of JAK2 and STAT3, which were all notably higher in PECs. By contrast, no increase was observed in the PI3K and AKT expression of both PECs and TECs. Western blot analysis of the control group, IL-6 group, sIL-6R group, and co-cultured IL-6 and sIL-6R group for 48 h were shown in Fig. 4. * P < 0.05; **P < 0.01

IL-6 or sIL-6R alone had no effect on the proliferation of ECs. Considering the predominant higher proliferation of ECs when co-culture with both IL-6 and sIL-6R for 48 h, we examined the effects of IL-6 or sIL-6R on the cell signaling pathways in EC proliferation for 48 h, in terms of the gp130, JAK2/STAT3 and PI3K/AKT expression. First, we found the administration of IL-6 or sIL-6R alone could not promote the expression of all the factors (Fig. 4), however, co-culture with both IL-6 and sIL-6R lead to a synergistic increase in gp130 expression, and this value was especially much higher in PECs with respect to the mRNA (RT-PCR, mean − △CT: −14.9 ± 0.22 *vs.* −19.5 ± 0.15 for IL-6/sIL-6R *vs.* control in PECs, *P* = 0.0005; −16.2 ± 0.11 *vs.* −18.4 ± 0.22 for IL-6/sIL-6R *vs.* control in TECs, *P* = 0.045) and protein levels by Western blot analysis (Fig. 4). Furthermore, phosphorylation of JAK2 and STAT3 in PECs were significantly increased when co-culture with both IL-6 and sIL-6R for 48 h, and the phosphorylation of both factors were not so obvious for 48 h in TECs, while phosphorylation of PI3K and AKT were not affected in both cells (Fig. 4).

## Discussion

This study reported that PECs demonstrated a much higher level of cell proliferation under co-culture with IL-6/sIL-6R by automatically up-regulating their cell surface gp130 proteins and increased phosphorylation of JAK2 and STAT3. The present study confirmed that hypoxia induced elevated IL-6 levels in PECs, however, no direct effects on cell proliferation were observed. Animal studies confirmed that peritumoral MVD was much higher than tumor MVD when tumors were large. Therefore, PECs and their associated peritumoral angiogenesis microenvironment are worthy of attention.

The novel finding in this study is that peritumoral angiogenesis was much higher than tumor angiogenesis when tumors had increased in size. The peritumoral angiogenesis microenvironment was much richer in terms of its much higher levels of hypoxia, IL-6, IL-6R, gp130, and CD68(+) macrophages. The present study showed that PEC proliferation relied on the up-regulation of IL-6/sIL-6R and gp130, which was in accordance with peritumoral hypoxia. Previous studies showed that anti-angiogenic factors exert an inhibitory effect on HCC growth mainly through anti-tumor angiogenesis [23, 28–31]. However, higher peritumoral angiogenesis is more important for intrahepatic metastasis during the progress of tumor growth. Attention should be given to peritumoral angiogenesis as the major therapeutic target.

In the present study, higher peritumoral IL-6 levels were proven to be derived from PECs. The IL-6 levels were induced by peritumoral hypoxia and associated with large tumor sizes. Therefore, we postulated that the compression effect on peritumoral tissues, as induced by the primary tumor, could create the peritumoral hypoxia environment, which contributed to IL-6 expression in peritumoral tissue and subsequent PEC proliferation.

IL-6 plays several roles in the pathway. Combining the membrane containing IL-6R and gp130 forms the IL-6/IL-6R/gp130 complex, which activates the JAK2/STAT3 or PI3K/AKT pathway and subsequent biological activities [19, 20], such as EC proliferation. Our present study found that IL-6 alone was unable to directly stimulate TEC and PEC proliferation. The lack of IL-6R on the surface of ECs suggested that IL-6 may exert its effect on ECs by combining with sIL-6R, and then it combines with the EC surface gp130 and ultimately activates the JAK2/STAT3 or PI3K/AKT pathway via the trans-signaling pathway. Our *in vitro* study confirmed that sIL-6R was an important factor of the vascular EC proliferation signaling pathway, which was consistent with other studies [21, 22], however, we also found that only the combination of IL-6 and sIL-6R could induce EC proliferation via JAK2/STAT3 signaling pathways however not the PI3K/AKT pathway. Previous studies have proven that macrophages or neutral granulocytes can contribute to sIL-6R secretion [26, 27, 32]. We found that the increased extensive distribution of macrophages was also associated with IL-6R. However, whether or not IL-6R is derived from macrophages requires further investigation.

In addition, we found that PECs had a stronger response to co-culture with IL-6 and sIL-6R compared with TECs. This result may be caused by the different cell signaling pathways between PECs and TECs during EC proliferation. gp130 is an important cell surface factor that is crucial for cell proliferation. Interestingly, we found that gp130 could be directly up-regulated in ECs after the administration of both IL-6 and sIL-6R. However, hypoxia had no direct effect on the expression of surface gp130 on PECs, which was in contrast to previous studies that stated that gp130 is controlled by HIF-1α [33, 34], and the controversial may be attributed to the fact that HIFs may differentially regulate the expression of the same angiogenic gene in different cell types; In our present study, the mechanism of gp130 expression unmodulated in PECs under hypoxia condition may be due to its distinct cell surface markers: IL-6R(−) and gp130 (+). In vivo study, only co-administration of both IL-6 and sIL-6R could lead to a synergistic increase in gp130 expression, which indicated that the up-regulation may be associated with effect of both IL-6 and IL-6R, and the elevated IL-6 on PECs by hypoxia could not alone up-regulate gp130 expression on PEC cell without IL-6R, and instead, the elevated peritumoral sIL-6R deriving from the macrophages together with elevated peritumoral IL-6 could lead to the higher expression of gp130 in peritumoral tissue, which had no direct correlation with peritumoral hypoxia.

The PECs comprise the key members of the peritumoral angiogenesis microenvironment. Previous studies suggested that the differences in the molecular phenotype of vascular ECs in normal and diseased tissues leads to different functions in both cell types [35]. This phenomenon was confirmed by our studies between PECs and TECs. The optimized immune magnetic sorting method allowed us to successfully obtain PECs with a high level of purity. These cells could be successfully passed in culture and used in the associated angiogenesis research studies [15, 36]. In the present study, the automatic up-regulation of gp130 as well as the increased phosphorylation of JAK2 and STAT3 in response to the combination of IL-6 and sIL-6R in PECs may also explain its more pro-angiogenic properties. PECs may be more sensitive to the anti-angiogenesis therapy target at IL-6/sIL-6R or gp130/JAK2/STAT3.

## Conclusions

This study demonstrated that PECs and TECs differed in their mode of angiogenesis. Although both cell types demonstrated proliferation only under co-stimulation with IL-6 and sIL-6R, the PECs showed a much higher proliferation rate, which was related to the automatic up-regulation of cell surface gp130 in PECs, as well as the increased phosphorylation of JAK2 and STAT3. In addition, the PI3K/AKT signaling pathways was not associated with the cells. Elevated peritumoral IL-6 expression in PECs, as well as peritumoral IL-6R expression induced by peritumoral hypoxia, could promote PEC proliferation and peritumoral angiogenesis. Furthermore, given the sensitivity of PECs to IL-6/sIL-6R, the PECs are the potential new targets for anti-angiogenic treatments.

## Additional file

Additional file 1: Table S1. Primers used for real time-PCR. (DOC 39 kb)

## Abbreviations

IL-6: Interleukin-6
sIL-6R: Soluble interleukin-6 receptor
PECs: Peritumoral endothelial cells
TECs: Tumor endothelial cells
HCC: Hepatocellular carcinoma
MVD: Microvessel density
wk: Weeks
EC: Endothelial cells
HUVECs: Human umbilical vein ECs
IHC: Immunohistochemistry
IOD: Integrated optical density
hr: Hours
CCK-8: Cell counting kit-8
TBS: Tris buffered saline
PBS: Phosphate buffered saline
RT-PCR: Reverse transcription-PCR
CoCl_2_: Cobalt chloride
