# Supplementary material for: Higher proliferation of peritumoral endothelial cells to IL-6/sIL-6R than tumoral endothelial cells in hepatocellular carcinoma
Source: BMC Cancer. 2015 Nov 2;15:830. doi: 10.1186/s12885-015-1763-2 (PMC4629315; doi:10.1186/s12885-015-1763-2)
Supplement: Additional file 1: Table S1. — Primers used for real time-PCR. (DOC 39 kb) [file 12885_2015_1763_MOESM1_ESM.doc]

***Supplementary Table 1 Primers used for real time-PCR***

| Gene name | Orientation | Primer sequence (5' - 3') | Species |
| --- | --- | --- | --- |
| IL-6 | forward | GAACTCCTTCTCCACAAGCG | human |
| IL-6 | reverse | TTTTCTGCCAGTGCCTCTTT | human |
| gp130 | forward | GGGCAATATGACTCTTTGAAGG | human |
| gp130 | reverse | TTCCTGTTGATGTTCAGAATGG | human |
| β-actin | forward | CATCTCTTGCTCGAAGTCCA | human |
| β-actin | reverse | ATCATGTTTGAGACCTTCAACA | human |
| IL-6 | forward | GACAAAGCCAGAGTCCTTCAGAGAG | mouse |
| IL-6 | reverse | CTAGGTTTGCCGAGTAGATCTC | mouse |
| IL-6R | forward | AAGAGTGACTTCCAGGTGCC | mouse |
| IL-6R | reverse | GGTATCGAAGCTGGAACTGC | mouse |
| gp130 | forward | CAGCAGCAGGTTTCAGATCAC | mouse |
| gp130 | reverse | GGACAAAGTCACACCTGGGC | mouse |
| β-actin | forward | CACGATGGAGGGGCCGGACTCATC | mouse |
| β-actin | reverse | TAAAGACCTCTATGCCAACACAGT | mouse |
